# Supplementary material for: Causal Relationship between Aging and Anorexia Nervosa: A White-Matter-Microstructure-Mediated Mendelian Randomization Analysis
Source: Biomedicines. 2024 Aug 16;12(8):1874. doi: 10.3390/biomedicines12081874 (PMC11351342; doi:10.3390/biomedicines12081874)
Supplement: Supplementary file 1 [file biomedicines-12-01874-s001.zip › Supplementary Figure/Figure S4/Singe SNP plot of MR analysis for TL to ubm-b-1594.pdf]

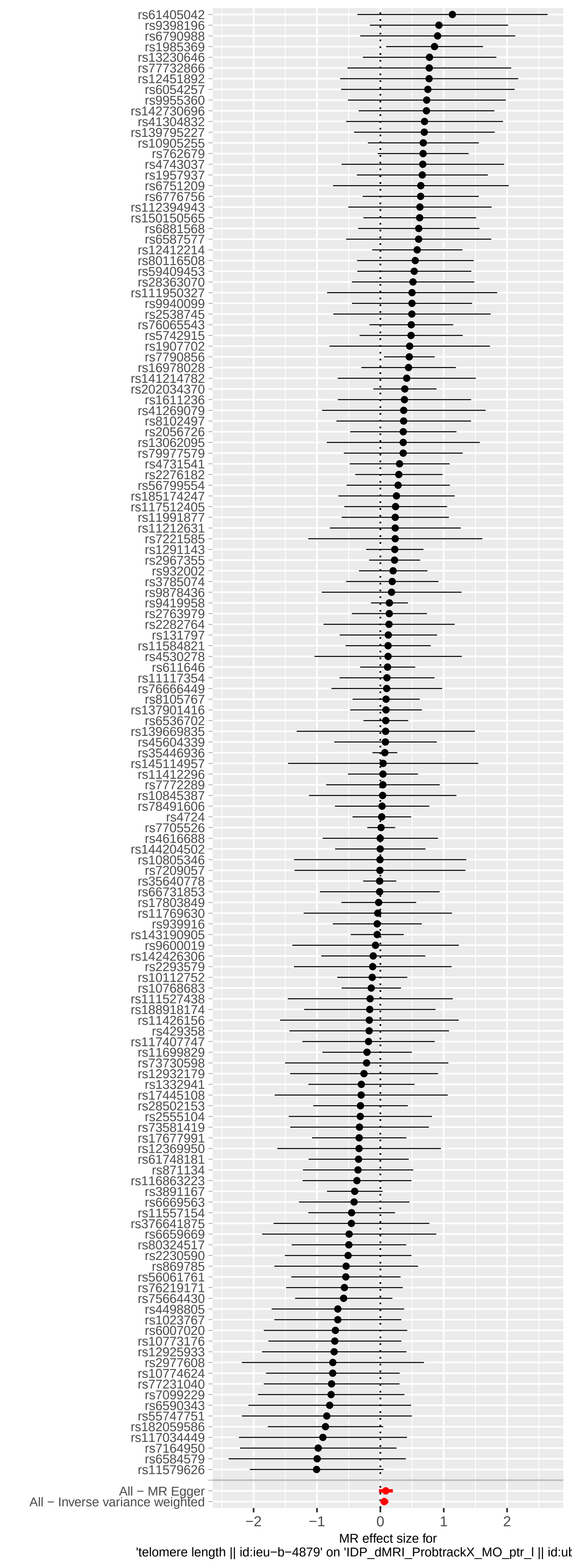
rs61405042
rs9398196
rs6790988
rs1985369
rs13230646
rs77732866
rs12451892
rs6054257
rs9955360
rs142730696
rs41304832
rs139795227
rs10905255
rs762679
rs4743037
rs1957937
rs6751209
rs6776756
rs112394943
rs150150565
rs6881568
rs6587577
rs12412214
rs80116508
rs59409453
rs28363070
rs111950327
rs9940099
rs2538745
rs76065543
rs5742915
rs1907702
rs7790856
rs16978028
rs141214782
rs202034370
rs1611236
rs41269079
rs8102497
rs2056726
rs13062095
rs79977579
rs4731541
rs2276182
rs56799554
rs185174247
rs117512405
rs11991877
rs11212631
rs7221585
rs1291143
rs2967355
rs932002
rs3785074
rs9878436
rs9419958
rs2763979
rs2282764
rs131797
rs11584821
rs4530278
rs611646
rs11117354
rs76666449
rs8105767
rs137901416
rs6536702
rs139669835
rs45604339
rs35446936
rs145114957
rs11412296
rs7772289
rs10845387
rs78491606
rs4724
rs7705526
rs4616688
rs144204502
rs10805346
rs7209057
rs35640778
rs66731853
rs17803849
rs11769630
rs939916
rs143190905
rs9600019
rs142426306
rs2293579
rs10112752
rs10768683
rs111527438
rs188918174
rs11426156
rs429358
rs117407747
rs11699829
rs73730598
rs12932179
rs1332941
rs17445108
rs28502153
rs2555104
rs73581419
rs17677991
rs12369950
rs61748181
rs871134
rs116863223
rs3891167
rs6669563
rs11557154
rs376641875
rs6659669
rs80324517
rs2230590
rs869785
rs56061761
rs76219171
rs75664430
rs4498805
rs1023767
rs6007020
rs10773176
rs12925933
rs2977608
rs10774624
rs77231040
rs7099229
rs6590343
rs55747751
rs182059586
rs117034449
rs7164950
rs6584579
rs11579626
All − MR Egger
All − Inverse variance weighted
−2
−1
0
1
2
MR effect size for
'telomere length || id:ieu−b−4879' on 'IDP_dMRI_ProbtrackX_MO_ptr_l || id:ub
